# Clinical validation of an integrated risk assessment test incorporating genomic and non-genomic data for sporadic breast cancer in Colombia

**DOI:** 10.3389/fgene.2025.1556907

**Published:** 2025-07-02

**Authors:** Harvy Mauricio Velasco Parra, Danny Styvens Cardona, Cesar Augusto Buitrago, Melisa Naranjo Vanegas, Sebastián Gutiérrez Hincapié, Carolina Jaramillo Jaramillo, Alicia Maria Cock-Rada, Carolina Benavides Duque, Clara Patricia Piedrahita, Catalina Bustamante, Leonel Andrés González Niño, Jen Kintle, Scott Kulm, Alessandro Bolli, Paolo Di Dominico, Giordano Botta, George B. Busby, Juan Pablo Valencia-Arango

**Affiliations:** ^1^ Personalized Medicine Group, Unidad de Bioentendimiento, Bioscience Center- Ayudas Diagnósticas SURA, Medellín, Colombia; ^2^ Data Science Department, Bioscience Center - Ayudas Diagnósticas SURA, Medellín, Colombia; ^3^ Omics Science Center, Unidad de Bioentendimiento, Bioscience Center – Ayudas Diagnósticas SURA, Medellín, Colombia; ^4^ Ayudas Diagnósticas Sura, Bioscience Center, Medellín, Colombia; ^5^ Clinical Research Group, Bioscience Center – Ayudas Diagnósticas SURA, Medellín, Colombia; ^6^ Medical imaging & AI in health SURA, Bioscience Center – Ayudas Diagnósticas SURA, Medellín, Colombia; ^7^ Allelica Inc., New York, United States

**Keywords:** polygenic risk score, breast cancer, risk stratification, prediction, epidemiology, Colombia

## Abstract

**Introduction:**

Breast cancer risk arises from a complex interaction of genetic, environmental, and physiological factors. Integrating Polygenic Risk Scores (PRS) with clinical risk factors can enhance personalized risk prediction, especially in diverse populations like Colombia.

**Objective:**

To evaluate the predictive performance of ancestry-specific PRS combined with clinical and imaging risk factors for breast cancer in Colombian women.

**Methods:**

We developed and validated ancestry-specific PRS using diverse genetic datasets. A cohort of 1,997 Colombian women, including 510 breast cancer cases (25.5%) and 1,487 controls (74.5%), were recruited. Clinical data, such as breast density and family history, were analyzed for predictive ability using the area under the receiver operating characteristic curve (AUC). Participants were categorized into genetic ancestry groups: Admixed American, African, and European. PRS were applied to the cohort and adjusted for clinical factors to assess risk prediction.

**Results:**

Breast density and family history were the strongest individual predictors, with AUCs of 0.66 and 0.64, respectively. Most participants were of Admixed American ancestry (70% of cases, 73% of controls). The combined PRS showed an Odds Ratio per Standard Deviation of 1.56 (95% CI 1.40–1.75) and an AUC of 0.72 (95% CI 0.69–0.74) when adjusted for family history. Incorporating PRS with clinical and imaging data improved the AUC to 0.79 (95% CI 0.76–0.81), significantly enhancing predictive accuracy.

**Conclusion:**

Combining ancestry-specific PRS with clinical risk factors provides a more accurate approach for breast cancer risk stratification in Colombian women. These findings support the development of precise, population-specific risk assessment models.

## Introduction

Cancer, including breast cancer, remains a leading cause of global mortality, driven by both inherited genetic predispositions and accumulated environmental exposures (Kocarnik et al., 2022; Bray et al., 2024). Polygenic inheritance describes the collective influence of numerous genetic variants and their interaction with environmental factors on traits, risk, or diseases, deviating from traditional Mendelian genetics (Crouch and Bodmer, 2020). Polygenic Risk Scores (PRS) estimate the cumulative effects of these common genetic variants on disease risk, offering a single metric that represents an individual´s genetic predisposition to a particular condition. PRS are derived from Genome-Wide Association Studies (GWAS), which examine the associations of millions of genetic variants with various diseases and traits (Visscher et al., 2017; Buniello et al., 2019). While individual Single Nucleotide Variants (SNVs) generally have modest effects, their combined influence can account for a significant proportion of heritability in many disorders (Wray et al., 2007). PRS have garnered attention as valuable tools for stratifying individuals into risk categories, thereby enabling targeted medical interventions.

Notably, the risk associated with PRS in the upper percentiles of a population distribution often mirrors well-established clinical risk factors, such as elevated LDL cholesterol for cardiovascular disease or high breast density for breast cancer (Busby et al., 2020; Mars et al., 2022; Mars et al., 2020; Levin and Rader, 2020; Ma and Zhou, 2021; Vachon et al., 2015; Cupido et al., 2021). Despite their promise, most PRS models are developed using datasets from individuals of European ancestry, creating challenges when applied to populations with different genetic backgrounds, such as Latinos (Lambert et al., 2019; Martin et al., 2019). Differences in allele frequencies, linkage disequilibrium patterns, and effect size may impact the predictive performance of PRS in non-European populations (Fatumo et al., 2022). Therefore, it is essential to validate PRS within the target populations for which they are intended, particularly those with diverse and admixed genetic ancestries (Gri et al., 2019; Park et al., 2017).

Breast cancer is the most prevalent malignancy among women worldwide, representing a quarter of all malignant tumors and the leading cause of cancer-related mortality in developing countries (Harbeck and Gnant, 2017). In Colombia, 17,018 new cases of breast cancer and 4,752 related deaths were reported in 2023, accounting for 14.5% of cancer incidence and 8.4% of cancer morality, respectively (World Health Organization, 2023). While reductions in breast cancer mortality have been observed in some countries due to improved screening and rapid therapeutic interventions, these strategies primarily focus on early detection and treatment rather than prevention (Viaña González, 2020).

Mammography remains a cornerstone for breast cancer screening, yet its role as a standalone risk assessment tool is limited. Incorporating genetic testing and other innovative methodologies can enhance the accuracy of risk prediction, aiding both prevention and early diagnosis. This underscores the need for integrated approaches combining genetic, clinical and imaging data to refine risk stratification and improve outcomes.

This study evaluates the clinical utility of combining five ancestry-specific PRS with clinical and mammographic data in a cohort of Colombian women with sporadic breast cancer. By focusing on a genetically diverse population, this research contributes to the growing evidence supporting the integration of PRS into clinical risk models, particularly in underrepresented populations such as Latin Americans. The findings aim to bridge the gap in precision medicine applications from non-European populations, with implications for improving breast cancer prevention and care in Colombia and beyond.

## Methods

### Study design and setting

This study was an observational case-control study, called “Soy Generación”, which included data collected between July and December 2022 in 5 cities in Colombia (Bogotá, Medellín, Barranquilla, Bucaramanga and Cali). The study included women (ages 40–80 years) with and without confirmed breast cancer diagnosis.

### Study population

The case group consisted of women (aged 40–65 years) who had been diagnosed with breast cancer during the study period and who were part of “Tiempo para ti” (Time for you–in English) program. This program offers integrated care for breast cancer patients through risk factor evaluation and clinical monitoring, provided by SURA Colombia.

The control group was composed of women (aged over 65 years old) who were insured by SURA Colombia, and who had no history of breast cancer. These patients were identified through negative mammography results. They were included to provide a comparison for assessing risk factors in a population without breast cancer, serving as a baseline for evaluating genetic and environmental influences on the development of the disease. Informed consent was obtained for all participants.

### Ancestry estimation and principal component analysis (PCA)

We applied a consistent ancestry estimation and PCA pipeline across all individual-level datasets used in this study, including PRS Training, Testing, and Validation cohorts. Genetic ancestry of individuals was estimated using 99,561 single nucleotide variants (SNVs) and the iAdmix method (Bansal et al., 2015), leveraging the 1000 Genomes Project reference dataset. Individuals were classified into five Super-populations: AFR (African), AMR (American), EAS (East Asian), EUR (European), and SAS (South Asian). Principal component analysis (PCA) was then performed by projecting individual-level genotype data onto a pre-computed PCA space derived from the 1000 Genomes Project dataset. This analysis yielded ancestry proportions from the five 1,000 Genomes Superpopulations and generated up to 10 principal components (PCs), which were used as covariates in GWAS analyses and downstream PRS modeling.

### Description of cohort used in PRS development

We employed a multi-stage study design integrating diverse population cohorts to develop, train, test, and validate ancestry-specific polygenic risk scores (PRS) for breast cancer. The foundation of PRS construction relied on three ancestry-specific genome-wide association studies (GWAS), which provided effect size estimates for score development. These included datasets representing East Asian, European, and African ancestries, derived from Biobank Japan (Sakaue et al., 2021; Michailidou et al., 2017), and the internally conducted Ghana Breast Health Study (Brinton et al., 2017), respectively.

Individual-level genotype data from five additional multi-ancestry cohorts were used for training and testing the PRS: the UK Biobank (UKB) (Bycroft et al., 2018), the Multi-Ethnic Study of Atherosclerosis (MESA, dbgap accession: phs000209. v2. p1) (Bild et al., 2002), the San Francisco Bay Area Latina Breast Cancer Study (SFBALCS, dbgap accession: phs000912. v1. p) (Michailidou et al., 2017), the Estrogen Receptor Negative Breast Cancer in African American Women study (CIDR, dbgap accession: phs000669. v1. p1) and High-Risk Breast Cancer GWAS (HRBC, dbgap accession: phs000929. v1. p1). These cohorts were selected for their diverse representation of ancestral backgrounds and phenotypic richness.

For external validation, we used an independent cohort of 1,997 admixed American women from SURA Colombia, selected based on clinical and demographic inclusion criteria to reflect the target population for clinical implementation.

### GWAS discovery datasets

GWAS summary statistics used for PRS construction were sourced from three datasets. Two were obtained from previously published studies: one from Biobank Japan, which included individuals of East Asian ancestry, and one from Michailidou et al. (2017), which included primarily European ancestry individuals. A third GWAS was conducted internally on the Ghana Breast Health Study cohort using the fastGWA-GLMM framework (Jiang et al., 2021; Nyante et al., 2019), applied to individual-level genotype data. For this African ancestry GWAS, a genetic relationship matrix (GRM) was computed based on approximately 100,000 linkage disequilibrium (LD)-independent variants, with age and the first four principal components of ancestry included as covariates.

Summary statistics underwent quality control and filtering steps prior to PRS development. Variants with minor allele frequency (MAF) ≤ 1% were excluded based on ancestry-specific allele frequencies from the 1000 Genomes Project. In the case of the largest European GWAS, fine-mapping was conducted using PolyFun (Weissbrod et al., 2020), with default parameters, with either one or ten causal variants selected per LD block and three different ancestry-specific LD maps (AFR, EUR, EAS) sourced from the PolyFun repository. Ancestry-specific LD maps were sourced from the PolyFun repository to improve the precision of effect size estimates. These processing steps yielded three ancestry-specific sets of GWAS summary statistics and six fine-mapped datasets, which were used in subsequent PRS construction. These filtering and fine-mapping procedures resulted in three sets of ancestry-specific summary statistics and six ancestry-specific fine-mapped datasets, which were subsequently used in the generation of PRS (Supplementary Table S1).

### Framework for PRS development

In this study, we used Allelica’s DISCOVER v1.3 software (Busby et al., 2023), a flexible PRS development platform that enables the integrated implementation of a range of existing polygenic score construction algorithms. For this analysis, we applied two distinct strategies: a single-ancestry PRS, developed using the stacked clumping and thresholding (SCT) method (Privé et al., 2019) method optimized in a European-ancestry Training dataset, and a trans-ancestry PRS, built using PRS-CSx (Ruan et al., 2022), a Bayesian approach that integrates GWAS summary statistics across multiple ancestral groups while accounting for linkage disequilibrium. DISCOVER generated a range of candidate scores, selected the best-performing PRS based on independent ancestry-specific Training datasets, and confirmed its predictive performance in separate Testing dataset. This dual framework, allowing for both population-specific optimization and improved cross-ancestry generalizability, is described in detail in the following sections.

### Single-ancestry PRS development

Due to its large sample size and increased statistical power, the European GWAS summary statistics were used to construct the single-ancestry polygenic risk score. We applied the SCT method (Privé et al., 2019; Ruan et al., 2022) within Allelica’s DISCOVER PRS development platform to generate a final panel of variants, risk alleles and effect sizes for downstream assessment. SCT generates –124,000 PRS panels by filtering the variants from the original summary statistics using a variety of different parameters related to Linkage Disequilibrium (LD) and significance thresholds. The resultant 124,000 different panels were then used in a penalized logistic regression to generate the optimal lineal combination of scores which maximizes outcome discrimination. The single-ancestry PRS training dataset, comprising both genotype and phenotype data, was used to optimize penalization hyperparameters.

### Trans-ancestry PRS development

To develop polygenic risk score (PRS) panels, we combined nine sets of prepared genome-wide association study (GWAS) summary statistics with the PRS-CSx (Ruan et al., 2022), algorithm (Supplementary Table S2), which was implemented using Allelica’s DISCOVER software. PRS-CSx utilizes a high-dimensional Bayesian regression framework that continuously shrinks the effect of each variant across multiple trans-ancestry GWAS. This allows for more accurate estimation of genetic risk by integrating information from various ancestry groups.

Given that PRS-CSx can simultaneously consider summary statistics from multiple ancestry groups to develop a consensus posterior effect, we employed several strategies to combine our available summary statistics. After combining the datasets, we ran the analyses with PRS-CSx using a range of hyperparameter values. In total, we generated 15 distinct PRS panels (Supplementary Table S2). For each GWAS combination, PRX-CSx was run 4 times varying the value of the global shrinkage parameter PHI (10^0^, 10^–2^, 10^–4^, 10^–6^), as suggested within PRSCSx documentation. All the other parameters were set to default values.

### PRS training

We utilized individual-level genotype data from five cohorts to train and test the PRS: UKB, MESA, HRBC, and SFBALCS, and CIDR. Details on the size and sources of these cohorts are provided in Table 1. Training of the single-ancestry PRS was performed in 77,994 European women from the first release of UKB. This allowed us to identify the best linear combination of 124,000 different panels, generated through a range of clumping/thresholding hyperparameters. The best performing ancestry-specific PRS among single- and trans-ancestry PRS was identified within the following cohorts: HRBC as European, MESA as multi-ancestry, and CIDR as African. Cohorts were combined together and best performing PRS were identified in each ancestry-specific subgroup. This approach allowed us to identify the most robust PRS for each population group. PRS performance were quantified by means of the PRS Odd ratio per Standard deviation (ORxSD) in logistic regression models with PRS as predictive variable, Brest Cancer as dependent variable, and age, the first four principal components of ancestry (PC1-4) and family history of Breast Cancer (when available in the dataset) as control variables.

**TABLE 1:** Datasets used to develop novel ancestry specific PRSs for breast cancer.

Cohort	Name	Country	Cases	Controls	Ethicity/Ancestry	Model development stage
BBJ	Biobank Japan	Japan	5,551	89,371	EAS	PRS Discovery
BCAC	Breast Cancer Association Consortium	Global	122,977	105,974	EUR	PRS Discovery
GHANA	Ghana Breast Cancer study	Ghana	869	1,618	AFR	PRS Discovery
CIDR	Centre for Inherited Disease Research	United States, Barbados, Nigeria	1,681	2,085	AFR	PRS Training^a^
MESA	Multi-Ethnic Study of Atherosclerosis	United States	76	2,947	AFR, EAS, EUR	PRS Training^a^
SFBALCS	San Francisco Bay Area Latinas Cancer study	United States	622	60	AMR	PRS Testing
HRBC	High Risk Breast Clinic	United States	2,343	2,059	EUR	PRS Training^a^
UKBB	United Kingdom Biobank	United Kingdom	143	2,607	AFR, EAS, SAS	PRS Training^a^
UKBB	United Kingdom Biobank	United Kingdom	6,269	71,725	EUR	PRS Training^b^
UKBB	United Kingdom Biobank	United Kingdom	14,641	164,309	AFR, EAS, EUR, SAS	PRS Testingt
Total			155,172	444,755		

^a^ Training dataset for single- and trans-ancestry PRSs.

^b^ Training dataset for single-ancestry PRSs.

### PRS testing

To assess the generalizability and predictive performance of the trained PRS, we used independent samples from the second release of UKB and the SFBALCS cohorts. Testing was conducted in ancestry-stratified subgroups corresponding to the populations included in the training step. PRS performances were quantified in each ancestry-specific group according to the same methodology described above. Age, PC1-4 and family history were used as control variables in the logistic regression models.

### PRS validation

For the Colombian validation dataset, we preselected 1997 patients from a total of 20,666 SURA Colombia affiliates based on adherence to specific inclusion criteria, which were validated using the company’s TERADATA SQL Assistant. Inclusion criteria included: females aged 40–80 years, living status, health conditions (including breast cancer), and mammography reports performed between 2017 and 2021. Additionally, breast cancer patients who met genetic testing criteria according to NCCN guidelines were tested with a Next-Generation Sequencing panel of 30 hereditary cancer genes. Patients with confirmed pathogenic/likely pathogenic variants were excluded. Of the 2,618 patients who met these criteria, 2055 consented to participate in the study. After validation of inclusion and exclusion criteria, 58 patients were excluded (see flowchart in Supplementary Figure S1). The final clinical validation cohort included 1997 patients: 1,487 controls and 510 cases. Baseline demographics and clinical data were collected from the electronic medical record (EMR) database.

DNA was extracted from blood samples collected from individuals in the validation cohort using the Applied Biosystems MagMAX DNA Multi-Sample Ultra 2.0 kit system, operated on the KingFisher Flex Magnetic Particle Processor 96DW platform. DNA quantity was determined using the Qubit 3 machine. Genotyping was performed using the Illumina Infinium HTS (High-Throughput Screening) Global Screening Array v3.0 (GSAv3.0) platform on the Illumina iScan Platform, capable of examining approximately 750,000 single-nucleotide variants (SNVs) and copy number variants (CNVs) per sample. The end-to-end analysis workflow was handled by the Biodiscovery NxClinical SW platform. Raw genotype data were imputed into a joint callset of 82,579,889 genome-wide variants using the BEAGLEv5.4 imputation tool and the 1000 Genomes Project v3 Imputation Reference Panel.

### Statistical analysis

Descriptive statistics (mean, median, percentiles, proportions) were used, with associations assessed using Fisher´s exact test or Chi-square (p < 0.05 considered significant). Differences between groups were tested using Student’s t-test, Mann-Whitney U, and Kruskal–Wallis tests, with normality assessed by the Shapiro-Wilk test. Polygenic score differences across ancestries were evaluated using Student’s t-test or Mann-Whitney U, as appropriate. Model applicability was assessed by calculating Odds Ratios (OR), standard deviation (SD), p-values, and the Area Under the Receiver Operating Characteristic (AUC) curve.

### Estimating risk

Polygenic risk scores (PRS) were calculated as the additive sum of effect sizes for risk alleles:

$$PRS=\sum_{i=1}^{n}\beta_{i}x_{i}$$

Where $\beta_{i}$ is the effect size (logarithm of the OR) for SNPi and $x_{i}$ is the allele dose (0, 1, or 2). PRS values were adjusted using principal components from the 1000 Genomes Project. Relative risk was estimated using the z-score of the PRS value in an ancestry-matched reference distribution:

$$Relative\;Risk=\exp\left(Z*\;\log\left(ORxSD\right)\right)$$

Relative risks were averaged across five PRS, weighted by ancestry proportions. After obtaining the relative risk values for each patient, we estimated the absolute 10-year risk of breast cancer using obtained epidemiological data for specific localities in Colombia (Medellin, Bogota, Cali, Barranquilla) collected for the purposes of this study (Supplementary Figure S2) and generated recalibrated risk curves for each using the approach of Darst et al. (2021). The approach constrains the PRS-specific absolute risks for a given age to be equivalent to the age-specific incidence for the entire population. Therefore age-specific incidence rates were calculated to increase or decrease based on the PRS category estimated risk and the proportion of the population within the PRS category. Note that both incidence and survival are a function of time (age) and are calculated for a given percentile of the PRS (*k*).

Absolute 10-year breast cancer risk was estimated using Colombian epidemiological data, recalibrated with risk curves based on Darst et al. (2021). Cumulative 10-year risks were calculated using the following formula (34):

$$AR_{k}\left(t\right)=\sum_{0}^{t}P_{ND}\left(t\right)S_{k}\left(t\right)I_{k}\left(t\right)$$

Where $P_{ND}\left(t\right)$ is the probability of survival from other causes, $S_{k}\left(t\right)$ is survival from breast cancer, and $I_{k}\left(t\right)$ is breast cancer incidence for risk category *k*.

### Reclassification methods

Reclassification was performed to evaluate the impact of integrating Polygenic Risk Scores (PRS) with clinical risk factors on breast cancer risk stratification. The reclassification process involved comparing the predicted risk categories before and after the inclusion of PRS. We used the Net Reclassification Improvement (NRI) and Integrated Discrimination Improvement (IDI) metrics to quantify the improvement in risk prediction. Participants were categorized into risk strata based on their predicted probabilities, and changes in these categories were analyzed to assess the effectiveness of the PRS integration. To further assess the impact of PRS on screening recommendations, we estimated the age at which each woman’s risk was equal to that of a 50-year-old woman, which is the current recommended age to start mammography screening in Colombia. This estimation was based on the PRS percentile assigned to each woman. We then compared this estimated age to the actual age of diagnosis for women diagnosed under the age of 50.

### Incorporating breast cancer PRS into risk models

Logistic regression was used to estimate effect sizes of risk factors, adjusting for ancestry and family history components. Discriminatory capacity was assessed using the ROC curve. The PRS model included:

$$Ca_{breast}=\alpha_{o}+\alpha_{1}PGS+\alpha_{2}edad+\alpha_{3}F_{1}+\cdots +\alpha_{k}PC_{1}+\cdots \alpha_{k+l}PC_{l}+\varepsilon$$

In the given context, $Ca_{breast}$ indicates a dichotomous variable (1: case, 0: control), $F_{i}$ represents the literature-reported factors associated with cancer, and $PC_{l}$ corresponds to the principal component l associated with ancestry.

In addition to evaluating single-ancestry and trans-ancestry PRS separately, we developed a combined PRS model by integrating the five ancestry-specific PRS into a weighted linear model. Weights were derived from the ancestry proportions estimated for each individual through iAdmix, as previously described. The combined PRS was calculated as a weighted average of each ancestry-specific PRS, where the contribution of each score was proportional to the individual´s inferred ancestry composition. This approach aimed to maximize predictive performance in admixed populations by leveraging information from multiple ancestries. The combined PRS was subsequently included as a predictive variable in logistic regression models, alongside age, principal components of ancestry (PC1-PC4), and family history of breast cancer. Discriminatory performance was assessed using the area under the received operating characteristic curve (AUC) and compared with single-ancestry and trans-ancestry models.

## Results

### Clinical and phenotypic characteristics

A total of 1,997 women were recruited for the study, of whom 510 (25.5%) were sporadic breast cancer cases, and 1,487 (74.5%) were assigned to the control group. Following laboratory-based revalidation of the data and variables collected during recruitment and project phases, clinical validation led to the exclusion of 21 individuals from the control group and 37 from the case group (Supplementary Figure S1). The mean age of the cases at the time of study entry was 55 years, whereas the age of the controls was 69 years. Clinical and phenotypic characteristics, known risk factors for breast cancer, were compared between cases and controls.

Significant differences were observed for several variables. Study entry weight was higher in cases than controls (median: 66 kg vs. 63 kg, p < 0.001), and age at menarche was slightly lower among cases (median: 12 vs. 13, p < 0.001). Duration of hormone replacement therapy (HRT) use also differed between groups (p < 0.0001), with higher proportion of controls having used HRT for more than 5 years. Conversely, no statistically significant differences were found for several reproductive factors, including history of at least one pregnancy, age at first delivery, age at menopause, or type of hormone replacement therapy regiment (Table 2).

**TABLE 2:** Clinical and Phenotypic characteristics of the patients in “Soy Generación” Study.

Characteristic	Case, N = 510^a^	Control, N = 1,487^a^	p-value^b^
Age	55 (49, 61)	69 (67, 71)	<0.001
BMI when diagnosed	26 (24, 30)	0 (0, 0)	NA
Study entry weight	66 (59, 74)	63 (56, 70)	<0.001
Age of menarche	13 (12, 14)	13 (12, 15)	<0.001
History of at least one pregnancy	433 (85%)	1,251 (84%)	0.7
Age at first deliver	24.0 (20.0, 28.0)	23.0 (20.0, 27.0)	0.3
Age at Menopause	48 (45, 51)	49 (45, 52)	0.2
Hormone Replacement Therapy			0.7
Estrogen treatment only	22 (81%)	19 (76%)	
Estrogen and Progestogen treatment	5 (19%)	6 (24%)	
Hormone replacement therapy			<0.001
2–5 years (%)	15 (56%)	3 (12%)	
Unknown	1 (3.7%)	3 (12%)	
More than 5 years	3 (11%)	16 (64%)	
Less than 2 years	8 (30%)	3 (12%)	
Breast Density Residual			<0.001
A	41 (8.0%)	302 (20%)	
B	218 (43%)	866 (58%)	
C	204 (40%)	288 (19%)	
D	47 (9.2%)	31 (2.1%)	

^a^ Median (IQR); Mean (SD); n (%).

^b^ Wilcoxon rank sum test; Welch Two Sample t-test; Pearson’s Chi-squared test; Fisher’s exact test.

^c^ BMI: body mass index.

Mammographic breast density exhibited a statistically significant relationship with breast cancer (p < 0.001) (see also Supplementary Table S3). The breast density distribution according to the ACR BI-RADS classification, showed a higher proportion of B, C and D types in the cases compared to the controls: 43% of cases were classified as type B compared to 19% of controls, 40% as type C of cases compared to 19% of controls, and 9.2% as type D of cases compared to 2.1% in controls (Table 2). When analyzing the impact of the clinical variables detected on their ability to discriminate for breast cancer, we observed areas under the curve ranging from 0.51 to 0.66, and for the multivariate model it was equal to 0.76. The variables of breast density and family history are the ones with the highest predictive power on their own with an AUC of 0.66 and 0.64 respectively.

### Development and testing of 5 ancestry-specific PRS for breast cancer

We evaluated 15 trans-ancestry PRS panels developed with PRS-CSx alongside a single-ancestry PRS across five genetic ancestry-specific cohorts. The best-performing PRS in each cohort was identified based on the highest Odds Ratio per Standard Deviation (ORxSD) (Table 3). The ORxSD ranged from 1.45 (1.31–1.77) for Allelica_BC_AFR_2022 in the African ancestry group to 1.75 (1.73–1.77) in the European ancestry group. The Allelica_BC_EUR_2020 PRS, previously developed using the Stacked Clumping and Thresholding algorithm (Patel and Khera, 2022), was the best-performing PRS in the European and East Asian ancestry groups. An ancestry adjustment and recalibration model revealed that 85% of study participants self-identified as admixed.

**TABLE 3:** OR*SD of the SURA Population and other populations using TARGET DATA.

Genetic ancestry	PRS	ORxSD	Total samples in testing dataset	Algorithm used and panel
AMR	Allelica_BC_AMR_2022	1.50 (1.17–1.93)	368	PRS-CSx - AFR-F10_AFR-G_EUR-F10_EUR-G (PHI 10^–4^)
AFR	Allelica_BC_AFR_2022	1.45 (1.31–1.60)	3,379	PRS-CSx - EAS-F1_EUR-F1 (PHI 10^–4^)
EUR	Allelica_BC_EUR_2020	1.75 (1.73–1.77)	172,588	SCT - single-ancestry PRS
SAS	Allelica_BC_SAS_2022	1.61 (1.43–1.84)	1,764	PRS-CSx -AFR-F1_EAS-F1 (PHI 10^–4^)
EAS	Allelica_BC_EUR_2020	1.71 (1.49–1.95)	1,219	SCT - single-ancestry PRS
SURA/Allelica	multiple	1.56 (1.40–1.75)	1,997	multiple

Details of polygenic risk scores derived from DISCOVER. The odds ratio per standard deviation (ORxSD) and total sample size of the Testing cohort are listed. The final row describes the performance of the above five ancestry-specific scores, combined using an ancestry-specific weighted average, across all individuals from the Colombia PRS, Validation cohort. For each ancestry-specific, the algorithm used and hyperparameter value is shown. See Supplementary Tables S1,S2 for further details.

**FIGURE 1:**
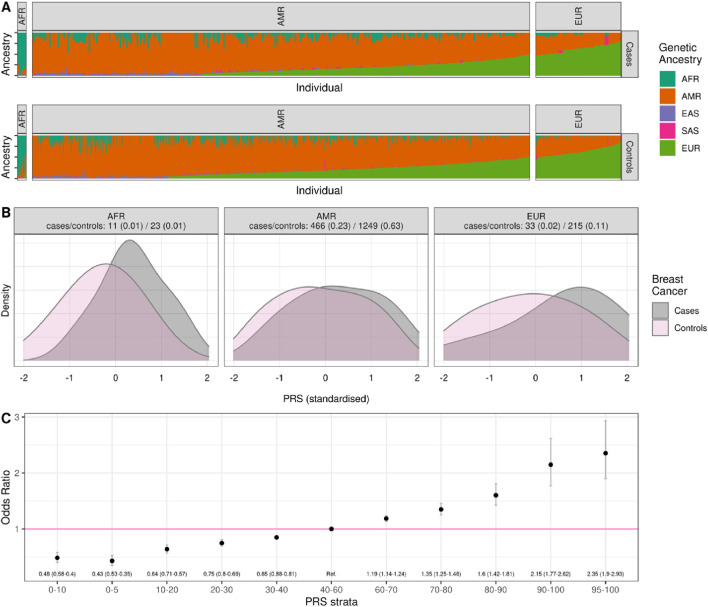
Distribution of genetic ancestries and PRS values in the Colombia PRS validation cohort by cases and controls. **(A)** Separately for cases and controls, we show the genetic ancestries present in 1997 individuals from Colombia grouped by major ancestry label; **(B)** the distribution of standardized PRS values in cases and controls, grouped by major ancestry label; **(C)** the Odds Ratio associated with being in a different strata of the PRS compared to the central quintile (40–60 percentile) based on a logistic regression controlling for the first 4 principal components of ancestry and family history.

Ancestry classification using iAdmix and 1000 Genomes Project superpopulations categorized participants into major ancestry groups (Figure 1A). Most individuals were classified as Admixed American (AMR) (84% of cases and 91% of controls), followed by European ancestry (14% of cases and 6% of controls). African ancestry was the least represented (1.5% of cases and 2.1% of controls). Significant differences in PRS distributions between cases and controls were observed across ancestry groups (Figure 1B; Supplementary Tables S4,S5).

### Predictive performance of the multi-ancestry PRS in the colombian population

The ORxSD of the multi-ancestry PRS applied to the Colombian population was 1.56 (95% CI: 1.39–1.74), indicating a 1.56-fold increased risk of breast cancer per standard deviation in the PRS. This result was consistent with values reported for European (1.75; 95% CI: 1.73–1.77) and African (1.45; 95% CI: 1.31–1.60) populations but exceeded those from smaller American cohorts (1.50; 95% CI: 1.17–1.93) (Table 3).

PRS values were used to estimate relative and absolute genetic risk. Women in the top decile of the PRS distribution had a 2.15-fold (95% CI: 1.77–2.62) increased risk compared to the reference quintile, while those in the highest 5% exhibited a 2.35-fold (95% CI: 1.90–2.93) increased genetic risk. These findings underscore the efficacy of PRS in stratifying individuals by genetic susceptibility to breast cancer (Figure 1C; Supplementary Table S6).

**FIGURE 2:**
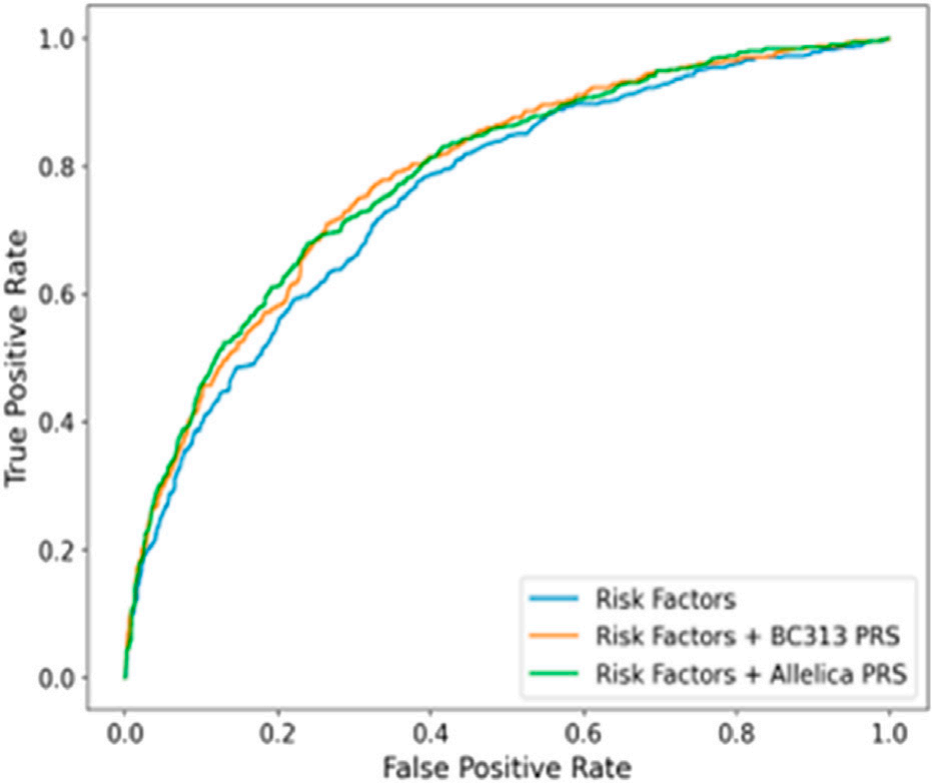
Figure 2. AUC graph of sura population compared to the prs model of Mavaddat et al. (Amir et al., 2010).

Adjusted for family history and ancestry, the AUC of the PRS was 0.72 (95% CI: 0.69–0.74). When combining clinical, imaging, and genomic data, the AUC increased to 0.79 (95% CI: 0.76–0.81), with genetic factors contributing approximately 8% to the prediction when family history alone was considered, and 3% when all factors were included (p < 1.4e-12) (Figure 2). Comparisons with datasets from other studies revealed similarities in PRS metrics (Supplementary Table S7). Despite a relatively modest AUC, PRS-based patient reclassification demonstrated clinical relevance, particularly when integrated with modalities like BI-RADS.

### Reclassification results

The reclassification analysis demonstrated significant improvements in risk prediction when PRS was integrated with clinical factors. The NRI indicated that 15% of participants were correctly reclassified into higher risk categories, while 10% were correctly reclassified into lower risk categories. The IDI showed an overall improvement in the discrimination ability of the model, with an increase in the AUC from 0.72 to 0.79. These results underscore the utility of PRS in enhancing breast cancer risk stratification.

PRS stratification categorized participants into four risk groups based on the percentile distribution in the control population: low-risk (<30th percentile), standard-risk (30th-69th), moderate-risk (70th-89th), and high-risk (≥90th percentile). Among breast cancer cases, 8.6% were classified as high-risk, 17.8% as moderate-risk, 33.7% as standard-risk, and 39.8% as low-risk. In the control group, 9.7% were high-risk, 12.8% moderate-risk, 31.3% standard-risk and 46.3% low-risk.

Among the 231 breast cancer cases diagnosed before age 50, 104 (45.0%) had an estimated screening eligibility age lower than their diagnosis age. On average, high-risk patients would have qualified for screening 15.2 years earlier, moderate-risk patients 8.4 years earlier, and low-risk individuals approximately 5.3 years later than the standard screening age of 50.

**FIGURE 3:**
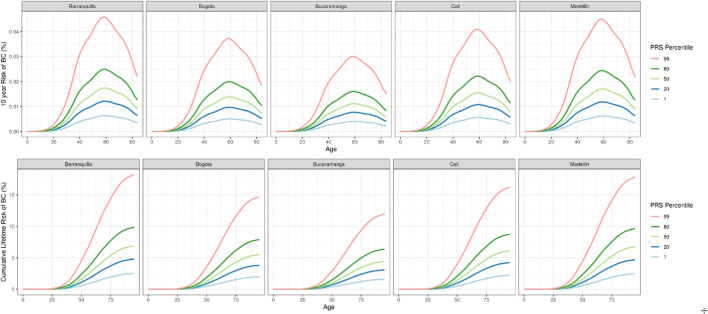
10 years and Cumulative lifetime risk of breast cancer based on region-specific incidence and mortality data and the effect size of the 5 ancestry-specific PRSs in Colombia.

**FIGURE 4:**
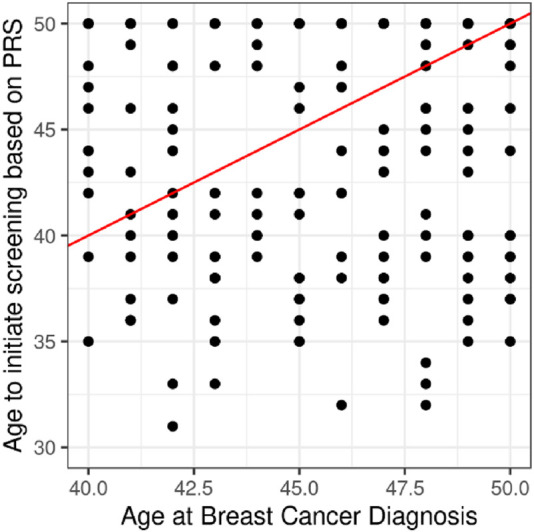
Projection of cases under 50 years of age. We applied the PRS to each woman to assign them a PRS percentile and from this we estimated the age at which her risk was equal to that of a 50-year-old woman and define this as the age to start screening. Of the 231 cases diagnosed under the age of 50, 104 (45.0%) have an estimated age of screening that is younger than the age of diagnosis.

Cumulative breast cancer risk was estimated based on observed incidence and mortality data across five Colombian regions (Figure 3). Risk was highest in Barranquilla and Medellín, with individuals in the highest PRS decile showing more than double the lifetime risk compared to those with average PRS values, and a sevenfold increased risk compared to the lowest PRS group (Figure 4). Ten-year risk peaked around age 60 in all regions.

## Discussion

Few studies have examined the effect of polygenic risk scores (PRS) in Latin American or Hispanic populations (Patel and Khera, 2022; Gaziano et al., 2016; Duncan et al., 2019; McArdle et al., 2021; Félix et al., 2023). The limited genomic characterization of these admixed populations poses challenges in validating PRS-based strategies, particularly for complex diseases such as breast cancer (Félix et al., 2023). This study provides clinical validation of PRS in a highly admixed Colombian cohort. Despite the diverse ancestry composition, the observed OR per standard deviation and AUC values suggest comparable predictive performance to European datasets, reinforcing the potential to extend genomic risk prediction to underrepresented populations (Sweeney et al., 2008; Martínez et al., 2017). Our findings were additionally supported by comparisons with external biobanks, showing consistent risk discrimination.

Multiple PRS studies have focused on breast cancer risk, largely in European and North American cohorts. Mavaddat et al. developed a PRS with 313 SNPs optimized for estrogen receptor (ER)-specific breast cancer, reporting an ORxSD of 1.61 (95% CI: 1.57–1.65) and an AUC of 0.630 (95% CI: 0.628–0.651) in a European cohort (Mavaddat et al., 2019). Despite including only 2.2% Hispanic participants, these results are comparable to our Colombian population. Similarly, Allman et al. observed improved ORxSD values when integrating 71 SNPs with the Gail or IBIS models in Hispanic women, findings corroborated in their later work (Allman et al., 2021; Allman et al., 2015). Shieh et al. demonstrated that integrating classical risk factors with PRS improved AUC values in Latin American women, aligning closely with our findings (Shieh et al., 2016; Shieh et al., 2020).

Triviño et al. reported PRS performance in a Spanish cohort, yielding an ORxSD of 1.41 (95% CI: 1.24–1.61) and an AUC of 0.8 (95% CI: 0.77–0.83), emphasizing ancestry-related nuances (Trivino et al., 2020). Ray et al. confirmed adequate discrimination of PRS-based models in a Colombian cohort, although without reporting overall ORxSD (Ray et al., 2017). Collectively, these studies highlight the robust predictive power of our PRS, which appears well-calibrated for the Colombian population.

Breast cancer prevalence differs across populations due to variations in reproductive behavior (Sweeney et al., 2008), socioeconomic factors (Martínez et al., 2017), and ancestry (Hines et al., 2017). The ancestry composition of our cohort aligns with meta-analyses of Latin Americans but differs from the higher European ancestry proportions reported by Ruiz et al. and Rodrigues de Moura et al. in the central regions of Colombia and Latin America (Ruiz et al., 2022; Rodrigues de Moura et al., 2015). Interestingly, Rey et al. found that Colombian women with higher American and lower European ancestry proportions had elevated odds of developing breast cancer (Rey-Vargas et al., 2022).

A significant risk of breast cancer is associated with germline pathogenic/likely pathogenic (P/LP) variants in high-or moderate-penetrance cancer-predisposing genes (Rojas and Stuckey, 2016). Although carrier status of P/LP variants was not systematically assessed across the entire cohort, individuals identified through NCCN clinical criteria were excluded from the analysis.

Other known risk factors for breast cancer include family history, particularly in first-degree relatives (Collaborative Group on Hormonal Factors in Breast Cancer, 2001), hormonal factors, lifestyle (Mcpherson et al., 2001), and breast density (Amir et al., 2010). All these variables, except the age of first birth, showed significant differences in women with breast cancer in our cohort. Alongside traditional risk prediction models such as BRCAPRO, Gail, BCSC, or Tyrer-Cuzick, there is growing interest in incorporating genomic risk factors like PRS to refine clinical risk stratification strategies.

The debate on optimal breast cancer screening strategies has shifted toward more personalized approaches. Wolfson et al. argued that PRS-based stratification may be more impactful than focusing solely on rare delirious variants or family history (Wolfson et al., 2021). Ho et al. demonstrated the superiority of combinatorial models that include PRS, family history, and deleterious variant detection, achieving a two-year AUC of 0.622 (0.608–0.636) (Ho et al., 2023; Morabia et al., 2003; He et al., 2006; Sims et al., 2008; Boora et al., 2016; Versmissen et al., 2015; Li et al., 2019; Kryukov et al., 2009).

Notably, PRS stratification identified significant proportions of women under 50 years of age who would benefit from screening–an improvement over Colombia´s current guidelines, which recommend mammography starting at age 50. High-risk patients in our cohort would have benefited from screening an average of 15 years earlier, with moderate-risk patients beginning screening approximately 8 years earlier. This underscores the value of PRS in personalizing screening strategies to better address early-onset cases. Our team is developing follow-up protocols tailored to patient risk levels, incorporating mammography, ultrasound, and MRI with annual monitoring starting at age 40. While these protocols align with international debates, further evaluation of clinical utility is underway (Ray et al., 2017; Eby, 2017).

The PRS presented here offers a significant advancement in breast cancer risk stratification for Colombian women. By integrating clinical, imaging, and genomic data, this model provides a comprehensive framework for personalized secondary prevention and anticipatory health management across diverse populations. Future work should prioritize the inclusion of underrepresented ancestries–such as Afro-Colombian and Indigenous populations–to improve the calibration and equity of PRS models in Latin America. Although most PRS are trained on European ancestry data, local validation is essential across all ancestral backgrounds presented in admixed populations such as Colombia (Bryc et al., 2010). These findings provide a foundation for incorporating genomic medicine into regional screening programs while paving the way for novel population management strategies and will help reduce disparities in predictive accuracy and ensure equitable implementation of genomic tools (Clarke and Thirlaway, 2011; Sweet et al., 2017; Roux et al., 2022; Brooks et al., 2021; French et al., 2020).

## Conclusion

This study provides a PRS and data consistent with findings from European populations, with clinical validation values demonstrating greater precision than several reports analyzing Latin populations, including those with Iberian or Colombian ancestry. At the time of this publication, it represents the most comprehensive Colombian case-control association study, with the best representation of the country’s multi-territoriality and multi-ancestry, focused on the clinical validation of a risk stratification test that integrates clinical, imaging, and genomic data. This unique model offers accurate risk stratification for sporadic breast cancer and holds promise for clinical applications in personalized secondary prevention, aiming to anticipate health management needs for patients and populations across Colombia.

## Limitations

Although this study focuses on analyzing the risk of sporadic breast cancer, there remains a possibility that some cases—clinically not meeting NCCN criteria for hereditary breast cancer—may carry pathogenic or likely pathogenic variants. Our PRS, based on an array platform, was not designed to detect such variants, representing a potential limitation in the scope of genetic information captured.

One limitation of our study is the variability in the completeness of family history information among participants. Approximately 30% of the participants had limited family history data, which may have impacted their classification as sporadic cases. This limitation should be considered when interpreting the results, as some individuals may have undetected genetic predispositions due to incomplete family history information. However, we now have comprehensive electronic medical records that include detailed family history information for first and second-degree relatives, with an average follow-up period of 5–8 years.

On the other hand, it is important to acknowledge that 55% of women diagnosed before age 50 would not have met the PRS-based threshold for early screening. This highlights a key limitation of current polygenic risk models and underscores the need for more comprehensive approaches. To address this, our team is actively working on the development of integrated models that combine PRS with clinical, imaging, and lifestyle factors to improve early detection, particularly in younger women. These efforts will be supported by future prospective studies aimed at validating the clinical utility and equity of such models in diverse Latin American populations.

While this is a clinical validation study, future research should evaluate qualitative and quantitative aspects related to the psychosocial impact on participants. This includes anxiety stemming from cancer risk stratification, concerns about perceived cancer risk, efforts to seek support and information, and behaviors associated with understanding and accepting risk information. Such evaluations are crucial to recognizing the broader consequences of these interventions at both personal and population levels. Additionally, expanding the sample size and including individuals with diverse ancestries, particularly Afro-Colombian and European populations, is necessary to enhance the predictive quality and accuracy of risk states. This is especially important given the known lower performance of predictive parameters in populations with African ancestry and Caribbean women compared to other groups.

## Data Availability

The data that support the findings of this study are available from the corresponding author upon reasonable request. Restrictions apply to the availability of these data due to ethical and privacy considerations.
