# Supplementary material for: Clinical validation of an integrated risk assessment test incorporating genomic and non-genomic data for sporadic breast cancer in Colombia
Source: Front Genet. 2025 Jul 2;16:1556907. doi: 10.3389/fgene.2025.1556907 (PMC12263362; doi:10.3389/fgene.2025.1556907)
Supplement: Supplementary file 1 [file Supplementaryfile1.docx]

**SUPPLEMENTARY MATERIAL**

**Supplementary Table 1.** The GWAS summary statistics, both taken directly from external sources and after post-processing conducted through the course of this investigation.

| **Ancestry** | **Name** | **Type** | **GWAS Source** |
| --- | --- | --- | --- |
| EUR | EUR-G | GWAS | Michailidou 2015 [26] |
| EUR | EUR-F1 | FINEMAPPING | generated from EUR-G 1 Causal SNP per LD block |
| EUR | EUR-F10 | FINEMAPPING | generated from EUR-G 10 Causal SNP per LD block |
| EAS | EAS-G | GWAS | Japan Biobank GWAS [25] |
| EAS | EAS-F1 | FINEMAPPING | generated from EUR-G 1 Causal SNP per LD block |
| EAS | EAS-F10 | FINEMAPPING | generated from EUR-G 10 Causal SNP per LD block |
| AFR | AFR-G | GWAS | Generated internally on Ghana Study [27] |
| AFR | AFR-F1 | FINEMAPPING | generated from EUR-G 1 Causal SNP per LD block |
| AFR | AFR-F10 | FINEMAPPING | generated from EUR-G 10 Causal SNP per LD block |

**Supplementary Table 2.** GWAS combination used in the PRS-CSx algorithm and corresponding PRS panel. For each GWAS combination, PRS-CSx generated four different PRS panel versions by scanning the PHI hyperparameter at values of 10^0^, 10^-2^ ,10^-4^, and 10^-6^. The number of SNPs reported for each GWAS combination is constant across PHI settings

| **PRS panel name** | **Summary Statistic Combination** | **N. SNPs** |
| --- | --- | --- |
| AFR-G_EUR-G | AFR-G + EUR-G | 194,737 |
| EAS-G_EUR-G | EAS-G + EUR-G | 249,977 |
| EAS-G_AFR-G | EAS-G + AFR-G | 207,815 |
| AFR-F10_EUR-F10 | AFR-F10 + EUR-F10 | 204,759 |
| AFR-F10_AFR-G_EUR-F10_EUR-G | AFR-F10 + AFR-G + EUR-F10 + EUR-G | 269,493 |
| AFR-F1_EUR-F1 | AFR-F1 + EUR-F1 | 191,145 |
| AFR-F1_AFR-G_EUR-F1_EUR-G | AFR-F1 + AFR-G + EUR-F1 + EUR-G | 256,735 |
| EAS-F10_EUR-F10 | EUR-F10 + EAS-F10 | 217,730 |
| EAS-F10_EAS-G_EUR-F10_EUR-G | EAS-F10 + EAS-G + EUR-F10 + EUR-G | 385,473 |
| EAS-F1_EUR-F1 | EUR-F1 + EAS-F1 | 205,595 |
| EAS-F1_EAS-G_EUR-F1_EUR-G | EAS-F1 + EAS-G + EUR-F1 + EUR-G | 319,656 |
| AFR-F10_EAS-F10 | AFR-F10 + EAS-F10 | 218,760 |
| AFR-F10_AFR-G_EAS-F10_EAS-G | AFR-F10 + AFR-G + EAS-F10 + EAS-G | 385,473 |
| AFR-F1_EAS-F1 | AFR-F1 + EAS-F1 | 205,433 |
| AFR-F1_AFR-G_EAS-F1_EAS-G | AFR-F1 + AFR-G + EAS-F1 + EAS-G | 374,424 |

**Supplementary Table 3.** Multivariate analysis of clinical characterization.

| **Model** | **AUC** | **AUC CI 2.5%** | **AUC CI 97.5%** |
| --- | --- | --- | --- |
| Residual Breast Density | 0.66 | 0.64 | 0.7 |
| Age at First Delivery | 0.51 | 0.47 | 0.53 |
| Age of Menopause | 0.55 | 0.52 | 0.58 |
| Age at Menarche | 0.58 | 0.56 | 0.61 |
| Hormone Replacement Therapy | 0.52 | 0.51 | 0.53 |
| Family History | 0.64 | 0.61 | 0.66 |
| Risk factors | 0.76 | 0.73 | 0.78 |

**Supplementary Table 4.** Ancestry distribution by cases and controls in the study

| **Ancestry** | **Mean AFR (SD)** | **Mean AMR (SD)** | **Mean EUR (SD)** |
| --- | --- | --- | --- |
| **Cases** | 6.2% (11.7%) | 70.0% (20.4%) | 22.9% (21.3%) |
| **Controls** | 8.1% (14.2%) | 73.7% (19.1%) | 16.5% (17.8%) |

The mean and standard deviation of ancestry components of individuals used in the analysis of the SURA datasets separated into three discrete ancestry groups.

**Supplementary Table 5**: Comparison of case and control densities considering ancestry.

|  | **z-score mean (SD)** | | **p-value** | |
| --- | --- | --- | --- | --- |
| **Pop** | **cases** | **controls** | **PRS means** | **PRS distribution** |
| **AMR** | -0.01 (0.98) | 0.27 (0.96) | 5.96×10^-11^ | 7.27×10^-12^ |
| **AFR** | -0.28 (0.70) | 0.38 (0.68) | 1.34×10^-2^ | 2.72×10^-2^ |
| **EUR** | -0.13 (1.06) | 0.57 (1.09) | 5.00×10^-4^ | 3.71×10^-4^ |

PRS Means and PRS distributions are statistically different for controls and cases in each genetic ancestry sub-population of the SURA dataset. We assessed statistical significance of the means by two-sided T test. We then assessed statistical significance of PRS distributions for Controls and Cases through a nonparametric test: The Mann-Whitney U test, a nonparametric statistical significance test for determining whether two samples are drawn from a population with the same distribution.

**Supplementary Table 6.** Odds Ratios associated with different strata of the PRS distribution.

| **Quantiles** | **OR (95% CI)** | **OR P-value** | **N. Cases** | **N. Controls** |
| --- | --- | --- | --- | --- |
| <5% | 0.04 (0.01-0.17) | < 0.005 | 2 | 98 |
| <10% | 0.26 (0.15-0.45) | < 0.005 | 19 | 181 |
| <20% | 0.48 (0.33-0.68) | < 0.005 | 63 | 337 |
| 20-40% | 0.67 (0.48-0.93) | < 0.005 | 85 | 314 |
| 60-80% | 0.9 (0.65-1.24) | 0.992 | 102 | 297 |
| >80% | 1.57 (1.15-2.13) | < 0.005 | 149 | 251 |
| >90% | 1.96 (1.35-2.84) | < 0.005 | 82 | 118 |
| >95% | 2.34 (1.4-3.92) | < 0.005 | 47 | 53 |

Odds Ratio (OR), 95% confidence intervals (CI 2.5% and CI 97.5%) and P-values for the association of PRS quantiles to Breast Cancer. OR were obtained in logistic regression models adjusted for the first four principal components of ancestry (PCA1-4) using 40-60% of PRS distribution as reference.

**Supplementary table 7.** Comparison of OR*DS and AUC between the sura model and Mavaddat et al.

| **Model** | **AUC** | **AUC CI 2.5%** | **AUC CI 97.5%** | **Brier Score** | **OR**  **PRS** | **OR PRS**  **CI 2.5%** | **OR PRS**  **CI 97.5%** | **OR PRS**  **P-value** |
| --- | --- | --- | --- | --- | --- | --- | --- | --- |
| **Mavaddat BC313 PRS** | 0.71 | 0.68 | 0.74 | 0.1683 | 1.53 | 1.37 | 1.71 | < 0.005 |
| **Sura Allelica PRS** | 0.72 | 0.69 | 0.74 | 0.1682 | 1.56 | 1.40 | 1.75 | < 0.005 |
| **Sura Allelica Risk factors** | 0.76 | 0.73 | 0.78 | / | / | / | / | / |
| **Risk factors + BC313** | 0.78 | 0.76 | 0.81 | 0.1509 | / | / | / | / |
| **Risk factors + Sura Allelica** | 0.79 | 0.76 | 0.81 | 0.1502 | / | / | / | / |

Area Under the Curve (AUC) and 95% CI for single risk factors and multivariate models. For models comprising any PRS, OR, OR CI, and OR p-values are reported. OR AUC and Brier Scores were obtained from logistic regression models. Logistic regression models comprising any PRS were adjusted for the first four principal components of ancestry (PCA1-4) and for Family History. The model defined as “Risk factors” combines all Breast Cancer clinical risk factors: Residual Breast Density, Age at First Delivery, Age of Menopause, Age at Menarche, Family History, and Hormone Replacement Therapy.

**Supplementary Figure 2:** Incidence and mortality of breast cancer in the population affiliated with sura Colombia


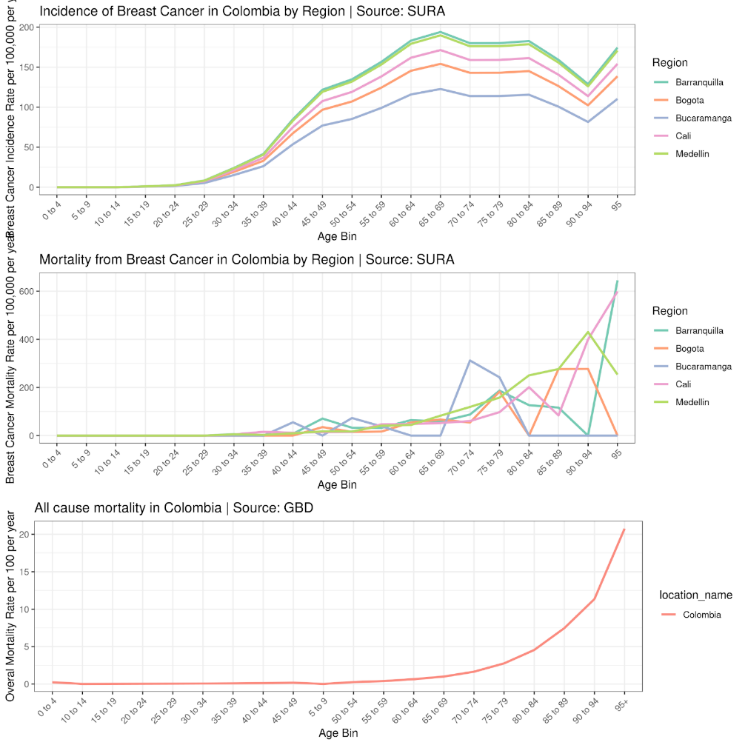


The incidence of breast cancer, mortality from breast cancer and all-cause mortality across a range of ages stratified by regions of Colombia.
